# Central Tendon Injury Impairs Regional Neuromuscular Activation of the Rectus Femoris Muscle

**DOI:** 10.3390/sports9110150

**Published:** 2021-10-27

**Authors:** Yoshiaki Kubo, Kohei Watanabe, Koichi Nakazato, Koji Koyama, Kenji Hiranuma

**Affiliations:** 1 Department of Judo Therapy, Faculty of Health Sciences, Tokyo Ariake University of Medical and Health Sciences, 2-9-1, Ariake, Koto-ku, Tokyo 135-0063, Japan; koyama@tau.ac.jp; 2 Laboratory of Neuromuscular Biomechanics, School of Health and Sport Sciences, Chukyo University, Toyota 470-0393, Aichi, Japan; wkohei@lets.chukyo-u.ac.jp; 3 Graduate Schools of Health and Sport Science, Nippon Sport Science University, 7-1-1, Fukasawa, Setagaya-ku, Tokyo 158-8508, Japan; nakazato@nittai.ac.jp (K.N.); hiranuma@nittai.ac.jp (K.H.)

**Keywords:** rectus femoris muscle, muscle strain injury, electromyography, physiology, sports medicine

## Abstract

We aimed to uncover which rectus femoris strain injury types affect regional activation within the rectus femoris. The rectus femoris has a region-specific functional role; the proximal region of the rectus femoris contributes more than the middle and distal regions during hip flexion. Although a history of strain injury modifies the region-specific functional role within the rectus femoris, it was not obvious which rectus femoris strain injury types affect regional activation within it. We studied 12 soccer players with a history of rectus femoris strain injury. Injury data were obtained from a questionnaire survey and magnetic resonance imaging. To confirm the region-specific functional role of the rectus femoris, surface multichannel electromyographic signals were recorded. Accordingly, eight legs had a history of central tendon injury, four had a history of myofascial junction injury, and four had a healed strain injury. When the injury was limited to the central tendon, the region-specific functional role disappeared. The region-specific functional role was confirmed when the injury was outside the central part. The neuromuscular function was also inhibited when the longitudinal range of the injured region was long. Our findings suggest that a central tendon injury with a long injury length impairs regional neuromuscular activation of the rectus femoris muscle.

## 1. Introduction

Regarding the neuromuscular function of the rectus femoris (RF), Watanabe et al. reported that the RF has a region-specific functional role, in which all regions of the RF contribute to knee extension (KE) force, while the proximal region contributes to hip flexion (HF) force [1]. Thus, the proximal and other (middle and distal) regions have different biomechanical functional roles [1]. Miyamoto et al. also reported a similar tendency of the neuromuscular function of the RF [2]. Moreover, this region-specific functional role of the RF has been confirmed during walking [3,4] and pedaling [5]. However, the region-specific functional role of the biceps femoris long head muscle has not been confirmed [4]. Thus, the RF has a more complex neuromuscular function than the biceps femoris long head muscle and plays an important role in RF function.

RF muscle strain injury is the most common quadriceps injury [6]. In particular, a central tendon injury is the most commonly encountered tear of the RF tendon [7]. The acetabulum is the origin of the central tendon. The central tendon continues distal to the acetabulum as a tendon within the muscle belly and appears as a muscle within a muscle. The central tendon continues for approximately two-thirds of the length of the muscle [8,9,10]. Central tendon injuries require protracted rehabilitation [6,11]. Wittstein et al. reported that most severe strain injuries to the RF involve the central tendon. While the majority of cases can be treated nonoperatively, some cases remain symptomatic and prevent a return to sports [12]. Balius et al. reported that the longer the length of the central tendon injury, the longer an athlete is unable to participate in sports [11]. Although central tendon injuries require attention, the reason why they require longer rehabilitation and are more severe is unknown. We speculated that the neuromuscular function might impact on the reason why they require longer rehabilitation. They are more severe because a muscle strain injury might impair the neuromuscular function [13,14,15,16].

Kubo et al. recently reported that the neuromuscular activation of the proximal region was reduced during HF in patients with a history of strain injury. Therefore, an RF strain injury might partly impact its neuromuscular function [15]. However, it was not obvious which injured part (i.e., central tendon, myofascial junction) affected RF neuromuscular function. Therefore, here we aimed to uncover the factors that impact the neuromuscular activation of the RF. We hypothesized that the neuromuscular function of the proximal region in the RF with a central tendon injury and/or long injury length would be significantly inhibited because these strain injuries sometimes have a worse prognosis. Revealing the neuromuscular function in the RF with a history of strain injury might contribute to the development of rehabilitation of RF strain injuries.

## 2. Materials and Methods

### 2.1. Experimental Design

This study added subjects to our previous study. Thus, a portion of the data was reported in the previous study [15]. The region-specific functional role was disturbed in the RF with a history of strain injury. In other words, there was no significant difference in the surface electromyography (SEMG) signal in the RF with a history of strain injury signal between the proximal region and middle region in hip flexion [15]. To confirm the region-specific functional role, we used the same methods that we have utilized in previous investigations [15]. Thus, the subjects performed an isometric maximal voluntary contraction (MVC) of KE and HF in both legs. During each MVC, multichannel SEMG signals from the RF were recorded. The injury site of the RF was diagnosed by a surgeon using magnetic resonance imaging (MRI). Furthermore, in this study, the noninjured RF was defined as the control RF.

First, we investigated whether there was a region-specific functional role of the entire RF with a history of strain injury. Second, we investigated whether there was a region-specific functional role of only RF with a history of central tendon injury. Third, we compared to the SEMG signal between RF with a history of a central tendon injury and noninjured RF to investigate the difference in both RFs in detail.

We also investigated the relationship with the SEMG signal and the longitudinal range of the injured region.

### 2.2. Subjects

All subjects were recruited from a football team belonging to the Japan University Football Association. First, we explained to the team coach the purpose of the study. Then, we asked the team coach to recruit as many players with a history of RF strain injury as possible during the off-season of 2015 and 2016. In total, twelve athletes with a history of RF strain injury (age, 19.6 ± 1.4 years; height, 172.5 ± 4.3 cm; weight, 65.0 ± 4.9 kg; sports-related experience, 13.3 ± 2.3 years) were enrolled in this study. The right leg was dominant in all subjects. In this study, the dominant leg was defined as the leg used to kick frequently. The Ethical Committee of Nippon Sport Science University approved the study protocol and the study (No. 015-H83, 26 October 2015). After the subjects were educated about the study purpose, potential risks, and protection of their rights, each provided written informed consent.

### 2.3. Questionnaire

Data of the participants’ physical characteristics and RF strain injury were collected through a questionnaire. The definition of RF strain injury was the same as in a previous study [15]. Thus, RF strain injury was defined as an indirect muscle injury that required medical attention and/or absence from subsequent training sessions or matches. Therefore, direct injuries such as contusion were not included as muscle strain injuries.

### 2.4. Magnetic Resonance Imaging

Studies of 0.3 T low-field MRI examinations of hamstring strain injuries have been reported [17]. Therefore, we used 0.3 low-field MRI with a 40-cm-diameter extremity coil in this study. The subject position was supine position. The field of view was 32 cm and the matrix was 256 × 256. Coronal T1-weighted images (reception time/echo time, 1000 ms/20 ms) were obtained. The scan range was 20 cm around the injury site that had been surveyed by questionnaire and the slice thickness was 10 mm. An orthopedic surgeon specializing in muscle injuries assessed the scans. The longitudinal range of the injured region (injury length) was calculated from an area that showed a low signal in MRI.

### 2.5. Multichannel SEMG Recording

Muscle strength was measured with a Biodex System 3 dynamometer (Biodex Inc., Shirley, NY, USA). The hip and knee were flexed at 90° and the trunk was fixed to the dynamometer. The subjects performed four tasks (MVC of KE and HF in both legs) after the warm-up and practice. As warm-up and practice, the subjects performed stretching of the hamstrings and quadriceps and more than three repetitions of concentric knee extension and flexion at 60°/s. The four tasks were performed at random. The subjects exerted isometric maximum force for 2 s in each trial. The subjects were given constant and identical verbal encouragement during each trial. In addition, at least 2 min of rest were allowed between each trial. The higher MVC torque was used in the multichannel SEMG analysis.

**Figure 1:**
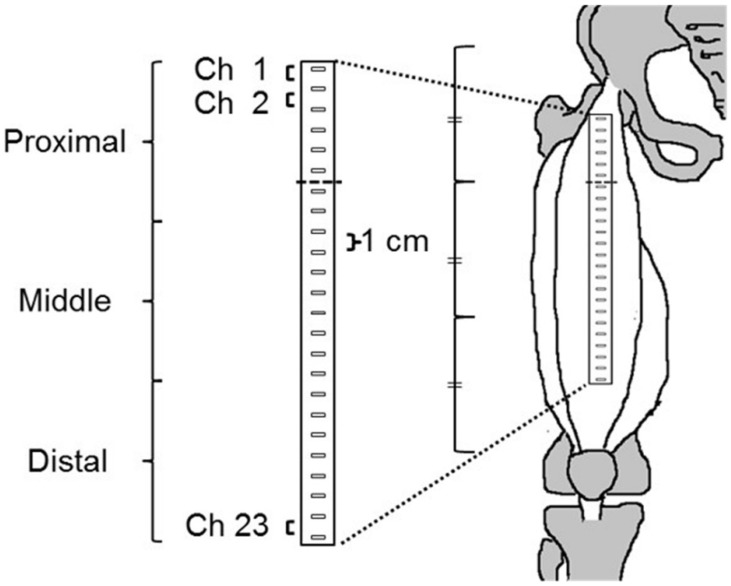
Locations of the 24 electrodes used to examine the rectus femoris muscle. The centers of the sixth and seventh electrodes from the proximal end were placed on the border of the proximal and middle region. Twenty-three bipolar surface electromyography signals were calculated from the electrode pairs between the neighboring electrodes and defined as Channels. Twenty-three channels were divided into three regions. The proximal region was composed of Ch1−7, the middle region was composed of Ch8−15, and the distal region was composed of Ch16−23. Ch, channel.

The region-specific functional role of the RF was demonstrated along a longitudinal line of the muscle [1,18]. Kubo et al. successfully recorded SEMG signals from the RF by using 24 electrodes arranged in one row and evaluated the region-specific functional role [15]. Therefore, during each task, SEMG signals from the RF were recorded using 24 electrodes arranged in a single row (inter-electrode distance, 10 mm; ELSCH16; OT Bioelettronica, Torino, Italy) (Figure 1).

The methods of SEMG recording were the same as in the previous study [15]. To determine electrode location, we confirmed for the edge of the superficial region of the RF using ultrasonography (SSD-3500; Aloka, Tokyo, Japan). In addition, marks were applied to the skin surface above the borders between the RF and adjacent muscle (i.e., vastus lateralis, vastus medialis, sartorius, and tensor fasciae latae muscles) using a waterproof pen. The RF had been surrounded by marks on the skin, so that the electrodes were attached within the markings after performing proper skin preparation. In addition, to proper skin contact with electrodes, conductive cream was inserted into the cavities of the electrodes. The electrodes were placed on the longitudinal axis of the RF that was defined as the previous studies [19,20]. The defined line was along a line between the anterior superior iliac spine and the superior edge of the patella. The centers of the sixth and seventh electrodes from the proximal end were placed at the proximal third of the longitudinal line of the RF (Figure 1). A reference electrode was placed at the head of the fibula.

Monopolar SEMG signals were amplified by a factor of 1000, sampled at 2048 Hz with an eighth-order Bessel band-pass filter at 10–500 Hz (anti-aliasing filter), and converted to digital form using a 12-bit analog-to-digital converter (EMG-USB 2; OT Bioelettronica, Italy). The recorded monopolar SEMG signals were transferred into analysis software (OT BioLab; OT Bioelettronica, Italy). In addition, 23 bipolar SEMG signals were calculated from the electrode pairs between the neighbor electrodes. Root mean square (RMS) values of each channel (Ch) were calculated from the bipolar SEMG signals that were sampled over 1 s epochs during the MVC. To investigate the region-specific functional role, 23 RMS values of the HF were normalized by those of the KE for each electrode pair. When the SEMG value of the HF was normalized by that of the KE, a region-specific functional role could be clearly observed [1,15]. In this study, normalized RMS was defined as HF/KE. The 23 HF/KE values were equally divided into three regions (Figure 1). We calculated the average HF/KE of the injured RF in each region.

### 2.6. Statistics

Data are expressed as the mean and standard deviation. After analyzing the variables for the normality distribution using the Shapiro−Wilk test, parametric or nonparametric statistical tests were used when appropriate, with a confidence level of 0.05. The HF/KE values among the three regions were compared using a one-way analysis of variance (ANOVA) or the Kruskal−Wallis test, followed by post hoc Bonferroni’s test. To compare two groups, Student’s *t* test or the Mann−Whitney U test was used. To investigate whether the injury length was correlated with the HF/KE, a single regression analysis was used. The mean HF/KE of Ch1 and Ch2 was used as the dependent variable. The injury length and the injury site (i.e., central tendon injury and the other injured part) were highly correlated on Pearson’s correlation coefficient (r = 0.76). Thus, the tendency showed that the subjects with a long injury length had a central tendon injury in this study. To avoid multicollinearity, the injury length was used as the explanatory variable.

*p* values < 0.05 were considered statistically significant. All statistical analyses were performed using the IBM SPSS Statistics 23 software for Windows (SPSS IBM; Japan Inc., Tokyo, Japan). In addition, the effect size (Cohen’s d, f) and statistical power were calculated by post hoc power analyses using the G*Power software (ver. 3.1; Heinrich-Heine-Universität Düsseldorf, Düsseldorf, Germany) when there was a significant difference in test statistic.

## 3. Results

### 3.1. Data of RF Strain Injury

Six athletes had a history of strain injury in the dominant leg, two athletes had a history of strain injury in the nondominant leg, and four athletes had a history of bilateral strain injury. Thereby, 16 of the 24 legs of the 12 athletes had a history of an RF strain injury. Eight control RF had no history of a strain injury. Eight RF strain injuries were induced by kicking and sprinting actions. All subjects were able to return to their pre-injury competitive level. However, five had complaints, i.e., tightness of the thigh, fatigability, occasional pain. The mean time from the initial injury to the study was 41.5 months (range, 12–96 months). Ten subjects underwent rehabilitation at different medical clinics, while two did not complete the rehabilitation. The mean duration of absence from practice or competition was 8.6 weeks (range, 1–24 weeks).

**Figure 2:**
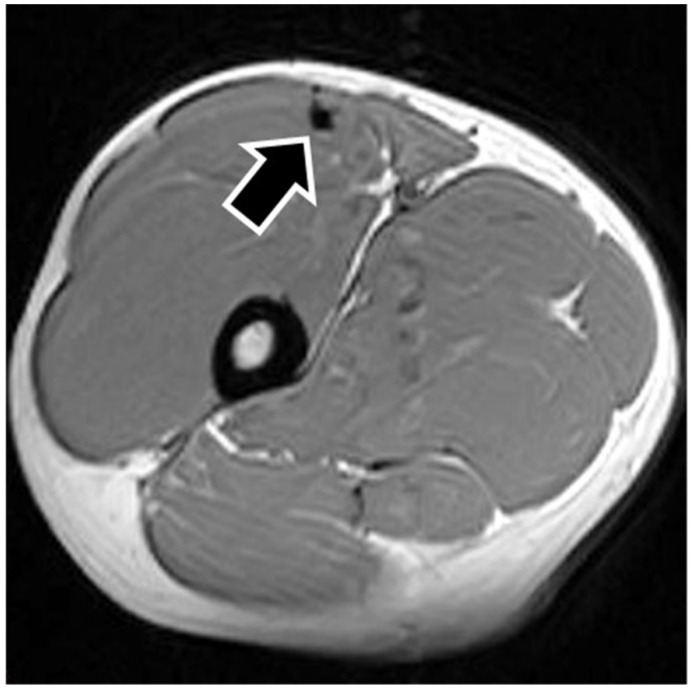
Pseudocyst of the central tendon. The figure shows an example of central tendon injury. The axial T1-weighted image shows a low signal surrounding the central tendon (black arrow), representing fibrous encasement.

**Figure 3:**
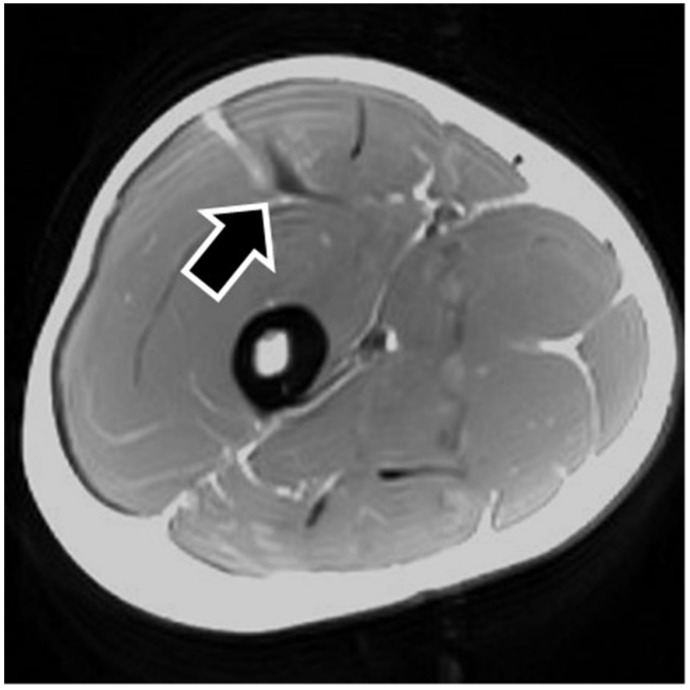
Injured rectus femoris muscle with a history of affecting the myofascial junction. The figure shows an example of myofascial junction injury. The axial T1-weighted image shows a low signal in the myofascial junction of the rectus femoris muscle (black arrow).

**Figure 4:**
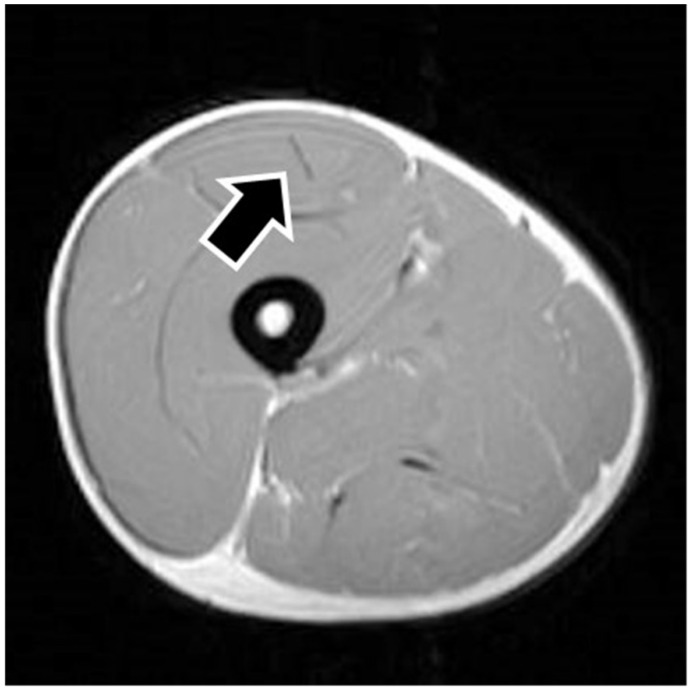
Noninjured rectus femoris muscle. The figure shows an example of noninjured rectus femoris muscle. The axial T1-weighted image does not show a low signal in the central tendon (black arrow).

MRI findings are shown in Table 1. In eight of the RF strain injuries, a low signal area was noted in the central tendon (Figure 2). In four of the RF strain injuries, a low signal area was noted in the myofascial junction of the RF (Figure 3). In four of the RF strain injuries, the axial T1-weighted image did not show a low signal (Figure 4). The longitudinal range of the injured region in the 12 RF that showed a low signal area in MRI was approximately 8.8 cm (range, 4–17 cm). When the injured part was limited to the central tendon, the longitudinal range of the injured region was approximately 10.5 cm (range, 5–17 cm).

### 3.2. Multichannel SEMG

The mean MVC torque of the right and left HF was 2.5 ± 0.4 Nm/kg and 2.6 ± 0.3 Nm/kg, respectively, while the mean MVC torque of the right and left KE was 3.8 ± 0.5 Nm/kg and 3.8 ± 0.4 Nm/kg, respectively. There was no significant difference in each comparison within each MVC torque.

Among the 16 RF with a history of strain injury, the HF/KE values of the proximal, middle, and distal regions were 0.98 ± 0.22, 0.69 ± 0.19, and 0.59 ± 0.19, respectively. One-way ANOVA showed a significant difference among three regions (*p* < 0.001, effect size = 0.86, statistical power = 0.99). In addition, by post hoc Bonferroni’s test, there was a significant difference between the proximal and middle region (*p* < 0.001) and the proximal and distal region (*p* < 0.001). Therefore, RF with a history of strain injury had a region-specific functional role.

When the injured site was limited to the central tendon, the HF/KE values of the proximal, middle, and distal regions in the eight RF were 0.91 ± 0.15, 0.72 ± 0.18, and 0.66 ± 0.18, respectively. The Kruskal−Wallis test showed a significant difference between the three regions (*p* = 0.034, effect size = 0.65, statistical power = 0.76). In addition, the Mann−Whitney U-test with Bonferroni correction showed a significant difference between the proximal and distal regions (*p* = 0.045). However, there were no significant differences between the proximal and middle regions (*p* = 0.15).

**Figure 5:**
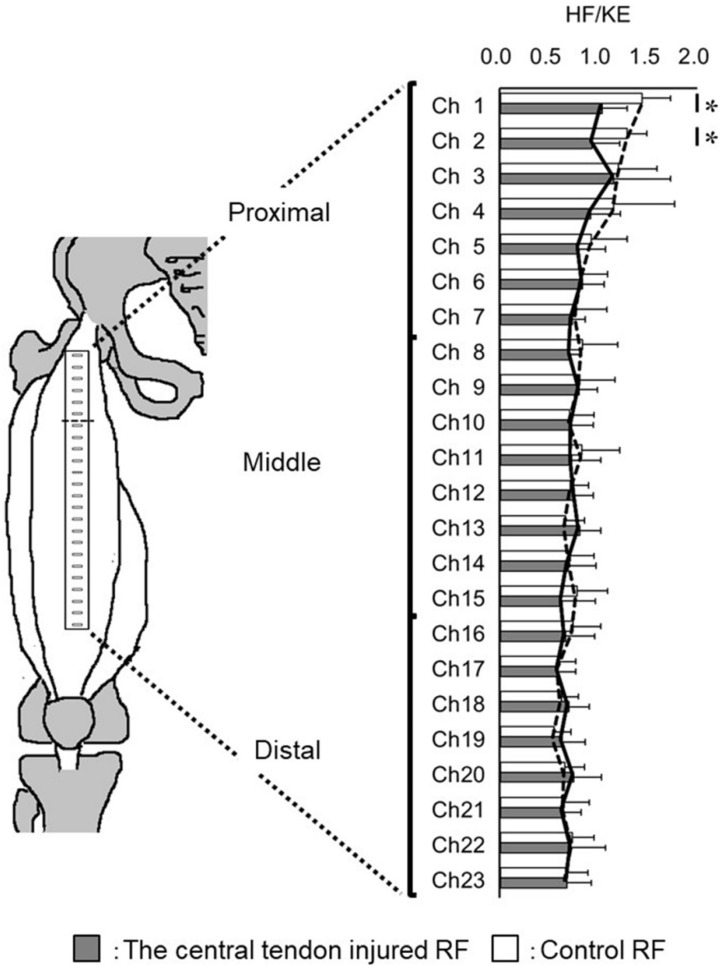
HF/KE of each channel. The solid line connects the mean HF/KE value in each channel of the central tendon injury of the RF. The dotted line connects the mean HF/KE value in each channel of the control RF. HF/KE, hip flexion/knee extension; RF, rectus femoris, * *p* < 0.05.

Furthermore, comparison of the RF with a history of the central tendon strain injury and control RF in each Ch of HF/KE revealed a significant difference in Ch1 (*p* = 0.01, effect size = 1.49, statistical power = 0.79) and Ch2 (*p* = 0.01, effect size = 1.48, statistical power = 0.79) of the HF/KE (Figure 5).

Among the control RF, there was a significant difference between the three regions (*p* = 0.002, effect size = 0.88, statistical power = 0.96). It was also confirmed that the control RF had a region-specific functional role (proximal vs. middle, *p* = 0.024; proximal vs. distal, *p* = 0.003).

To investigate the relationship between HF/KE and the longitudinal range of the injured region, single regression analysis was used. In this study, Ch1 and Ch2 of HF/KE in the central injured RF were significantly lower than those in the control RF (Figure 5). Thus, the mean HF/KE of Ch1 and Ch2 was used as the dependent variable. As a result, the mean HF/KE values of Ch1 and Ch2 were 1.28 when the longitudinal range of the injured region was 0 cm. This value decreases by 0.025 (95% confidence interval, −0.05 to −0.001) with each 1 cm increase. The adjusted R^2^ value was 0.21.

Detailed information on the questionnaire, MRI findings, and SEMG data is provided in the supplementary material (Tables S1–S3).

## 4. Discussion

The MRI of a grade 1 strain is of a feathery appearance. Hemorrhage and fluid around the central tendon greatly suggest an acute grade 2 strain. In chronic or healing RF strains, there may be a fibrous encasement of the central tendon, characterized on MRI as T1 and T2 linear low signal around or adjacent to the central tendon [7]. The MRI showed a low signal area in this study so that the central tendon injured RFs were speculated as grade 2.

In this study, when the injured part was limited to the central tendon, the region-specific functional role disappeared; however, when the injured part was not limited to the central tendon, the region-specific functional role was confirmed. In the previous report [15], the central tendon injury might have significantly affected the results because two-thirds of the injured RF involved the central tendon injury. Therefore, not all RF strain injuries might impact the region-specific functional role, but the central tendon injury impacted the region-specific functional role. The results were consistent with the fact that the central tendon injury requires a longer rehabilitation duration and is a most severe strain injury [6,11,12]. Furthermore, the neuromuscular function was inhibited when the longitudinal range of the injured region was long. The longitudinal range of the injured region is associated with rehabilitation period duration [11]. Although a prospective study is needed, the results of this study suggested that the inhibition of the neuromuscular function may be correlated with rehabilitation period duration and condition severity.

Regarding the effect of the central tendon injury on the neuromuscular activation, the HF/KE of the proximal region (i.e., Ch1 and Ch2) in the central tendon injury was significantly lower than that of the control. A decline in HF/KE indicates a decline in the contribution to HF effort because the SEMG signal of the HF was normalized by that of the KE. In particular, the proximal region in the RF contributes to the HF [1]. In addition, at high contraction intensity, central locus activations were located at the proximal region during HF compared to those at low contraction intensities [1]. Thus, the results of this study concluded that the proximal region contribution to the HF was inhibited by the central tendon injury. This suggested that reductions in the SEMG signal of the strain injured muscle may indicate that the previously strained muscle is unable to withstand the same amount of stress before failure compared with the noninjured muscle [16,21]. In an RF strain injury, the most proximal and anterior surface of the strain-injured RF might be regulated to reduce tension within the entire RF [15]. However, the reason why the central tendon injury inhibited the contribution to the HF is unclear because as far as we are aware, no study to date has investigated the muscle fiber contraction dynamics of RF. Although it remains a matter of speculation, Bordalo-Rodrigues et al. mentioned that the direct head is involved in the initiation of the HF, whereas the indirect head (the origin of the central tendon) plays a greater role during flexion [7]. Although further studies of the muscle fiber contraction dynamics of the RF are needed, the results of this study that HF/KE of the proximal region in the central tendon injury was inhibited and other injured parts were not inhibited can support the theory of these previous studies.

The longer longitudinal range of the injured region revealed that the HF/KE of the proximal region was inhibited. In this study, the longitudinal range of the injured region was evaluated for low signal areas on MRI. This area is considered incompletely healed because Hasselman et al. found that chronic RF strain injuries consist of highly vascular, fibrotic loose connective tissue surrounding the deep tendon with a serous fluid collection between the tendon and connective tissue, thus creating a pseudocyst [8]. The results of Hasselman et al. might support the finding that an injured muscle is unable to withstand the same amount of stress before failure than the noninjured muscle [16,21]. In addition, Hasselman et al. speculated that, with the chronic strain injury, the indirect and direct heads of the proximal tendon begin to act independently, creating a shearing phenomenon in contrast to what occurs in the normal RF [8]. Changes in the muscle contraction mechanism might inhibit the neuromuscular activation in the proximal region. In addition, the longitudinal range of the injured region might be correlated with inhibition of the neuromuscular activation because the HF/KE of the proximal region with the long injury length was more inhibited.

In this study, the proximal region of the HF/KE in subjects with a central tendon injury and/or long injury length was inhibited. Kouzaki et al. reported that the nerve conduction velocity of the hamstring-injured limb was significantly lower than that of the uninjured limb, although no significant side-to-side differences were seen in the control group [14]. However, all subjects in this and the previous studies [14,15] returned to their pre-injury competitive level. Thus, it was suggested that muscle strain injury causes nerve injury and then changes the neuromuscular function. Silder et al. reported that many athletes with a history of hamstring strain injury are possibly returning to sport with residual atrophy of the biceps femoris long head and/or hypertrophy of the biceps femoris short head and that it is possible that long-term changes in the musculotendon structure following injury alter the contraction mechanics during functional movement [22]. Therefore, when there was great inhibition in HF/KE, rehabilitation might require a long period to alter the contraction mechanics. Although further studies are needed, the evaluation of the neuromuscular function might be an index for predicting the required rehabilitation period.

As one prevention strategy for RF strain injury, Mendiguchia et al. recommended a greater use of concentric hip flexor strengthening exercises during the preseason [23]. We also suggest a greater use of concentric hip flexor strengthening exercises during the rehabilitation of central tendon injury and/or long injury length because the proximal region contributed most to HF inhibition.

This study has several limitations. First, although injury severity differed between the affected parts, we investigated the SEMG signal on the same level of the longitudinal line as the injured parts without considering severity. In other words, the electrodes for SEMG were not always placed just above the injured parts. The region-specific functional role of the RF was confirmed by the longitudinal line of the muscle, while the transverse line of the muscle does not affect the region-specific functional role of the RF [1,18]. Thus, there was no significant effect of injury severity on the main results. Second, this study was retrospective. Regarding the self-reported retrospective injury data, the findings were likely to be very accurate. However, requesting for further details, e.g., rehabilitation protocol, occurrence during a match or practice, would reduce the validity of the information provided [24]. We confirmed the RF strain injury by MRI and not only by the self-reported questionnaire. However, it was difficult to obtain injury details. To acquire further findings of the relationship between strain injury and the neuromuscular function, a prospective study is needed. Third, the number of subjects was small. We studied 16 RF injuries. The effect size ranged from medium to large. However, the statistical power was insufficient. Therefore, further studies are required. It was unclear whether the injured part or the injury length had a greater effect on the neuromuscular activation. Although the adjusted value of R^2^ in the injury length analysis was low, the neuromuscular activation of the central tendon injury with a long injury length was strongly inhibited in this study.

Although this study was basic and further studies are required, our findings suggest that inhibition of the neuromuscular function may be correlated with rehabilitation duration.

## 5. Conclusions

Here we investigated which factors affect the region-specific functional role of the RF. We found that the region-specific functional role was disturbed in central tendon injuries. However, the injured RF within other parts was confirmed to have a region-specific functional role. The longitudinal range of the injured region might also affect the neuromuscular function. Therefore, not all RF strain injuries impact the neuromuscular functions, but a central tendon injury with a long injury length inhibited the neuromuscular function.

## Figures and Tables

**Table 1:** MRI findings.

Player	Dominant Leg	Injured Side	Injured Part	Injured Region	Injury Length (cm)
A	R	R	no signal	-	-
B	R	R/L	central tendon/central tendon	p-m/m	17/5
C	R	R	myofascial junction	p-m	6
D	R	L	central tendon	p-m	9
E	R	R	myofascial junction	m	4
F	R	L	central tendon	p-m	10
G	R	R	central tendon	p	6
H	R	R/L	central tendon/no signal	m-d/-	13/-
I	R	R	no signal	-	-
J	R	R/L	central tendon/no signal	p-m/-	12/-
K	R	R/L	central tendon/myofascial junction	m-d/p-m	12/7
L	R	R	myofascial junction	p-m	5

R, right; L, left; p, proximal region; m, middle region; p-m, to middle region from proximal region; m-d, to distal region from middle region.

## Data Availability

The data presented in this study are available in the insert article and Supplementary Material here.

## Supplementary Materials

The following are available online at https://www.mdpi.com/article/10.3390/sports9110150/s1, Table S1. Physical characteristics of each subject. Table S2. SEMG data. Table S3. Average KE/HF and injury length of each subject
